# Peripheral blood inflammatory score using a cytokine multiplex assay predicts clinical outcomes in patients treated with atezolizumab-bevacizumab for unresectable HCC

**DOI:** 10.3389/fimmu.2025.1578422

**Published:** 2025-06-11

**Authors:** Hee Sun Cho, Soon Kyu Lee, Ji Won Han, Jung Hyun Kwon, Soon Woo Nam, Jaejun Lee, Keungmo Yang, Pil Soo Sung, Jeong Won Jang, Seung Kew Yoon, Jong Young Choi

**Affiliations:** ^1^ The Catholic University Liver Research Center, College of Medicine, The Catholic University of Korea, Seoul, Republic of Korea; ^2^ Department of Internal Medicine, College of Medicine, Seoul St. Mary’s Hospital, The Catholic University of Korea, Seoul, Republic of Korea; ^3^ Department of Internal Medicine, College of Medicine, Eunpyeong St. Mary’s Hospital, The Catholic University of Korea, Seoul, Republic of Korea; ^4^ Department of Internal Medicine, College of Medicine, Incheon St. Mary’s Hospital, The Catholic University of Korea, Incheon, Republic of Korea

**Keywords:** hepatocellular carcinoma, atezolizumab plus bevacizumab, prognostic score, biomarker, cytokine

## Abstract

**Background:**

Several serum cytokines have been proposed as biomarkers for predicting the outcomes of patients with hepatocellular carcinoma (HCC) receiving tyrosine kinase inhibitors. However, their role in atezolizumab plus bevacizumab (AB) treatment needs to be more elucidated.

**Methods:**

We examined various serum cytokines, including interferon-γ (IFN-γ), interleukin-10 (IL-10), IL-12, IL-17, IL-2, IL-6, and tumor necrosis factor, using a Luminex cytokine multiplex assay before AB treatment in prospectively enrolled 116 AB-treatment patients for the derivation cohort and 54 patients for the external validation cohort. We collected baseline characteristics, including neutrophil-lymphocyte ratio (NLR) and C-reactive protein (CRP) levels, and prospectively observed clinical outcomes.

**Results:**

Among various peripheral blood inflammatory markers, high NLR, CRP, IL-2, and IL-12 levels were significantly associated with poor progression-free survival (PFS) and overall survival (OS) in patients with AB-treated HCC. Through sensitivity analysis, we defined the high peripheral blood inflammatory score (PBIS) group, which included two or more of the following elevated factors: NLR, CRP, IL-2, and IL-12. The high PBIS group had elevated serum inflammatory cytokines and a higher tumor burden than the low PBIS group. A high PBIS score was an independent risk factor associated with poor OS, PFS, and objective response rate (ORR) in multivariate analyses, which was also confirmed in the validation cohort and propensity score-matched cohort. However, it was not a significant factor for OS, PFS, or ORR in lenvatinib-treated patients.

**Conclusion:**

These results suggest that a peripheral blood marker-based scoring system can significantly predict clinical outcomes in patients with AB-treated HCC. This non-invasive biomarker is expected to be a potential predictive and prognostic factor for AB treatment.

## Introduction

According to the global cancer statistics for 2020, hepatocellular carcinoma (HCC) is the third leading cause of cancer-related deaths and the sixth most frequently diagnosed cancer worldwide. (1) Advanced-stage HCC is categorized as tumors with portal vein tumor thrombosis (PVTT) or extrahepatic spreading (2) and for unresectable HCC, systemic therapy is the first choice of treatment, as with the introduction of sorafenib, an oral multi-tyrosine kinase inhibitor, in 2007 (3).

Atezolizumab is a drug targeting programmed death-ligand 1 (PD-L1), which enhances anti-tumor immunity by preventing PD-L1-mediated suppression of T-cell activity. Bevacizumab is an angiogenesis inhibitor targeting vascular endothelial growth factor (VEGF), which blocks tumor angiogenesis, thereby inhibiting tumor growth and metastasis. In the pivotal phase III IMbrave150 trial, atezolizumab plus bevacizumab (AB) demonstrated significantly improved median overall survival (OS; 19.2 vs. 13.4 months, p<0.001) and median progression-free survival (PFS; 6.8 vs. 4.3 months, p<0.001) compared with sorafenib, establishing AB therapy as the standard first-line systemic treatment for unresectable HCC. (4, 5) Since AB treatment was officially approved as the first-line treatment for unresectable HCC in 2020, the real-world data showed comparable but debatable outcomes. (6, 7) With accumulating results, investigating biomarkers to predict clinical outcomes in AB treatment is crucial for focusing its strength.

Several biomarkers from blood parameters, tissue markers, and imaging studies have been examined to predict the therapeutic outcomes of AB combination therapy. Data from blood samples included inflammatory markers, tumor markers, immune cells, and cytokines. (8, 9) They are easily obtainable in the clinical field and offer practical advantages over other biomarkers. Regarding AB treatment, blood biomarkers such as alpha-fetoprotein (AFP), vascular endothelial growth factor (VEGF), neutrophil-to-lymphocyte ratio (NLR), C-reactive protein (CRP), and albumin have been investigated. (8) Recently, studies have reported that higher baseline levels of serum IL-6 are associated with poor clinical outcomes of AB treatment, suggesting that serum cytokines may serve as biomarkers for AB treatment in HCC. (10) Nevertheless, as with multiple cohorts undergoing AB treatment, it is necessary to study and verify the cytokines related to their diversity.

Cytokines are signal-carrying proteins involved in cell-to-cell interactions that are soluble in the bloodstream, making them easily detectable. (11) As immune modulators, either immunosuppressive or immunostimulatory, they are involved in regulating immune cell activity and the formation of tumor cells. (12) Cytokines encompass a wide variety of soluble signaling proteins, including interleukins (ILs) and interferons (IFNs), each exhibiting distinct and diverse biological activities. ILs constitute a large family of cytokines primarily involved in regulating immune and inflammatory responses by modulating cell proliferation, differentiation, activation, and migration of immune cells. IFNs, classified into type I, II, and III, mainly mediate antiviral, antiproliferative, and immunomodulatory effects, playing critical roles in innate and adaptive immunity. (13) They are also involved in the clinical stages of HCC and exert distinctive and crucial effects on the tumor microenvironment. (13) Distinguishable cytokines can act as biomarkers to predict the outcomes of systemic treatment. Biomarkers such as IL-6, angiopoietin-2, TGF-β, and VEGF are expected to have prognostic value in systemic therapy for HCC. (13)

In this study, we investigated the potential of various cytokines in predicting the outcomes of AB treatment in patients with unresectable HCC. Cytokine levels in the serum collected from a prospective cohort were measured using a Luminex cytokine array. Their associations with clinical outcomes, such as OS, PFS, and objective response rate (ORR), were examined. We explored whether cytokines in combination with established markers, such as NLR and CRP, could serve as predictive markers for AB treatment.

## Patients and methods

### Study cohort

Ethical approval for this study was obtained from the Institutional Review Board of the Catholic University of Korea (approval number: KC22EASI0342), and the study was conducted following the principles of the Declaration of Helsinki. We prospectively recruited 116 patients with unresectable HCC receiving AB treatment at Seoul St. Mary’s Hospital as the derivation cohort, and 54 patients receiving AB treatment at Incheon St. Mary’s Hospital as the external validation cohort. Additionally, 21 patients receiving lenvatinib treatment at Seoul St. Mary’s Hospital were included as a comparative reference group to evaluate biomarker specificity. Patients aged ≥18 years with unresectable HCC treated with AB, with adequate hepatic function (Child-Pugh class A or compensated B7), Eastern Cooperative Oncology Group (ECOG) status 0-1, and available baseline cytokine assay results obtained within one week before treatment initiation were prospectively enrolled from May 2020. Patients who did not undergo baseline cytokine assays or lacked sufficient follow-up data for response evaluation were excluded. Clinical outcomes were observed for up to 4 years or until the date of last follow-up. Informed consents were obtained from all participants. Serum samples were collected before initial treatment.

### Cytokine multiplex assay

Cytokine concentrations were quantified using the MILLIPLEX MAP human cytokine/chemokine panel (Millipore, Germany) on a Luminex 200 instrument (Luminex, USA) following previously established protocols. (14)

### Assessment of clinical outcomes

Radiological and laboratory data were collected at the time of enrollment. Regular imaging was conducted every 3–4 cycles of AB, and treatment efficacy was monitored using the modified Response Evaluation Criteria in Solid Tumors. (2) OS and PFS were measured from the commencement of treatment to death, last follow-up, or disease progression. The ORR included “complete” and “partial” responses, while the disease control rate encompassed complete response, partial response, and stable disease.

### Statistical methods

Statistical analyses were performed using the R statistical software (version 4.0.3; R Foundation Inc., Austria) and SPSS (version 23.0; IBM Corp., USA). Continuous variables were compared using the Student’s t-test, and categorical variables were analyzed using the chi-square test. Propensity score matching with one-to-one nearest-neighbor matching within a 0.20 caliper width was applied to mitigate baseline differences between groups. Kaplan–Meier estimates were used for survival analyses, and Cox regression modeling was used to determine survival outcome predictors. Logistic regression was used to identify the response determinants. Statistical significance was set at p<0.05.

## Results

### Correlations between baseline characteristics and serum cytokine concentrations

Baseline clinical characteristics of patients in both the derivation and validation cohorts are summarized in **Table 1**. The two cohorts were generally similar in demographic and clinical profiles, including age, gender distribution, tumor burden, and liver function parameters. Using Spearman’s rank correlation analysis, we first measured the strength of the association between the serum concentrations of various cytokines and core baseline characteristics among all patients (**Supplementary Figures S1**, **S2**). Serum interferon-gamma (IFN-γ) levels positively correlated with intrahepatic tumor size (r = 0.199, p<0.05). Additionally, IL-10 positively correlated with poorer Child-Pugh score (r = 0.187, p<0.05), and serum levels of tumor markers, including AFP (r = 0.208, p<0.05) and protein induced by vitamin K absence-II (PIVKA-II) (r = 0.227, p<0.05). Moreover, it showed stronger correlations with intrahepatic tumor size (r = 0.376, p<0.001). IL-12 was positively correlated with ECOG (r = 0.190, p<0.05), AFP (r = 0.247, p<0.05) and intrahepatic tumor size (r = 0.230, p<0.05), and IL-6 was positively correlated with intrahepatic tumor size (r = 0.217, p<0.05). IL-2 and tumor necrosis factor levels were not associated with the clinical characteristics. These findings suggest that serum cytokine levels might be elevated in patients with high tumor burden.

**Table 1:** Patients’ characteristics.

	Derivation cohort (n=116)	Validation cohort (n=54)
Male gender	93 (80.2%)	46 (85.2%)
Age, years	63.0 [55.0;71.0]	63.0 [58.0;74.0]
BMI, kg/m^2^	23.7 [21.7;26.0]	23.7 [21.7;26.6]
Viral etiology	81 (69.8%)	44 (81.5%)
Treatment-naive	46 (39.7%)	30 (55.6%)
Total bilirubin, mg/dL	0.7 [0.5;1.2]	1.1 [0.6;1.5]
Albumin, g/dL	3.8 [3.5;4.1]	3.7 [3.3;3.9]
INR	1.1 [1.0;1.1]	1.1 [1.1;1.2]
AFP, ng/mL	552.0 [12.9;15845.1]	330.9 [8.0;4587.6]
PIVKA-II, mAU/mL	2105.5 [228.5;13583.0]	2566.4 [117.7;11755.0]
Largest intrahepatic tumor size, cm	5.5 [2.6;11.0]	8.1 [3.0;14.0]
Multiple intrahepatic tumors	82 (70.7%)	50 (92.6%)
PVTT	70 (60.3%)	38 (70.4%)
EHS	61 (52.6%)	29 (53.7%)
ECOG 0	76 (65.5%)	38 (70.4%)
irAEs	33 (28.4%)	10 (18.6%)
Objective responses	31 (26.7%)	14 (25.9%)

Data are presented as n (%) or median [interquartile]. BMI, body mass index; INR, international normalized ratio; AFP, alpha-fetoprotein; PIVKA-II, protein induced by vitamin K antagonist-II; PVTT, portal vein tumor thrombosis; EHS, extrahepatic spread; ECOG, Eastern Cooperative Oncology Group; irAE, immune-related adverse events.

### Development of the peripheral blood inflammatory score

To identify the peripheral blood inflammatory markers associated with clinical outcomes, including PFS and OS, we first conducted univariate Cox regression analyses of cytokines, NLR, and CRP (**Supplementary Table S1**). The analyses showed poor OS and PFS were significantly associated with elevated NLR and CRP, IL-12, and IL-2 levels. The optimal cut-off values for NLR, CRP, IL-12, and IL-2 were determined using Cox regression. Briefly, p-values from the log-rank test were calculated for multiple cut-off candidates, and the cut-off value with the lowest p-value was selected as the optimal point. As a result, cutoff points for each marker were determined as follows: NLR≥3.5; CRP≥0.13 mg/dL; IL-12≥11.6 pg/mL; and IL-2≥3.2 ng/mL. Using these markers, each assigned a value of 1 point, we generated the PBIS score, which ranged from 0 to 4 points. With the sensitivity analysis using Cox-regression, PBIS≥2 had the lowest p-values for OS and PFS, which was associated with poor PFS (HR = 2.10, CI = 1.31–3.36, p = 0.002) and OS (HR = 3.67, CI = 1.77–7.63, p = 0.001) (**Table 2**). We classified patients who met the above criteria into the PBIS-high (PBIS^hi^) group and the others into the PBIS-low (PBIS^lo^) group.

**Table 2:** Sensitivity analysis using univariate Cox-regressions for identifying an optimal cutoff point of peripheral blood inflammatory score.

	OS		PFS	
	HR (95% CI)	p	HR (95% CI)	p
PBIS≥1	7.48 (1.02-54.57)	0.047	2.87 (1.24-6.65)	0.014
**PBIS≥2**	**3.67 (1.77-7.63)**	**0.001**	**2.10 (1.31-3.36)**	**0.002**
PBIS≥3	2.45 (1.23-4.90)	0.011	1.87 (1.08-3.24)	0.025
PBIS≥4	1.95 (0.69-5.51)	0.207	1.79 (0.78-4.14)	0.173

OS, overall survival; PFS, progression-free survival; HR; Hazard Ratio; CI; Confidence Interval; PBIS, peripheral blood inflammatory score. Bold values indicate the lowest p-values for OS and PFS, which we defined high peripheral blood inflammatory score.

### Characteristics of the PBIS^hi^ group

We further analyzed whether the PBIS^hi^ and PBIS^lo^ groups, as defined above, could represent the overall systemic serum cytokine levels and concentrations of serum cytokines (IFN-γ, IL-10, IL-12, IL-17, IL-2, IL-6, and TNF) in AB-treated patients (**Supplementary Figure S3**). As a result, all cytokines were significantly higher serum concentrations as PBIS increased (p<0.0001), suggesting that high PBIS levels may be significantly associated with systemic inflammation. In addition, the PBIS^hi^ group showed higher AFP levels, intrahepatic tumor size, and a higher percentage of multiple tumors, suggesting that the PBIS^hi^ group may have a higher tumor burden than that in the PBIS^lo^ group (**Supplementary Table S2**).

### PBIS as a both predictive and prognostic marker

**Figure 1:**
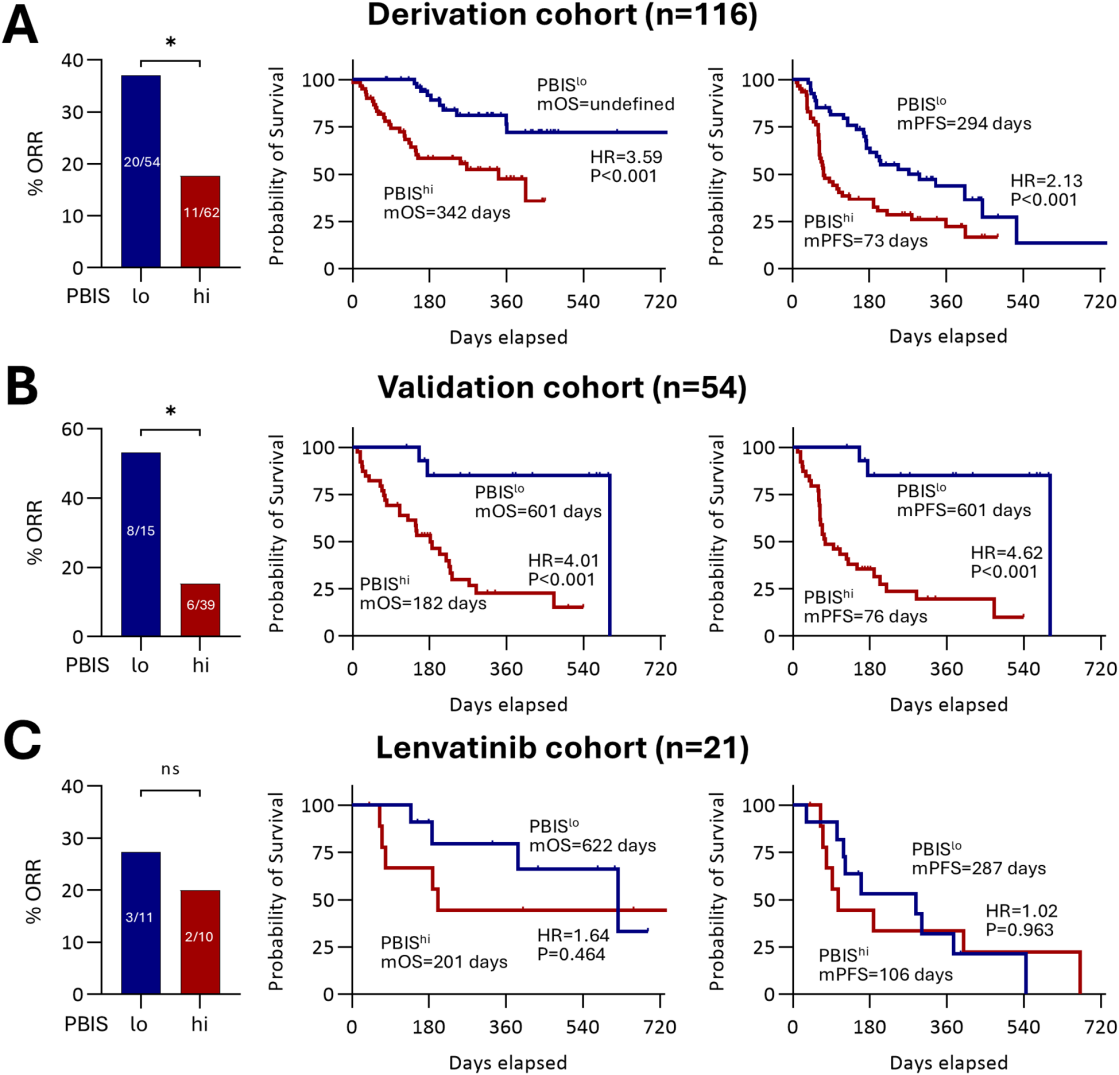
Implications of PBIS in clinical outcomes. The percentage of ORR, and survival curves regarding OS and PFS comparing PBIS^lo^ and PBIS^hi^ groups among **(A)** derivation cohort (n = 116, p<0.05), **(B)** validation cohort (n = 54, p<0.05), and **(C)** lenvatinib cohort (n = 21, p>0.05). ORR, objective response rate; PBIS, peripheral blood inflammatory score; mOS, median overall survival; HR, hazard ratio; mPFS, median progression-free survival. * p < 0.05. ns, non significant.

To test the clinical relevance of PBIS, we compared ORR, OS, and PFS between the PBIS^hi^ and PBIS^lo^ groups in the derivation cohort and validation cohorts (**Figure 1**). Baseline characteristics of each cohort are presented in **Supplementary Table S2**. In the derivation cohort, the PBIS^hi^ group showed a significantly lower ORR than that in the PBIS^lo^ group (11/62, 17.7% vs. 20/54, 37.0%; p = 0.022) (**Figure 1A**). In addition, the PBIS^hi^ group had poorer outcomes regarding OS (HR = 3.59, p<0.001) and PFS (HR = 2.13, p<0.001) than those in the PBIS^lo^ group (**Figure 1A**). Correspondingly, a lower ORR in the PBIS^hi^ group than that in the PBIS^lo^ group (6/39, 15.4% vs. 8/15, 53.3%, p = 0.012) was observed in the validation cohort (**Figure 1B**). Furthermore, the poorer OS (HR = 4.01, p<0.001) and PFS (HR = 4.62, p<0.001) in the PBIS^hi^ group were consistent with those in the derivation cohort (**Figure 1B**). However, the lenvatinib cohort revealed no significant differences in the ORR, OS, or PFS between the PBIS^hi^ and PBIS^lo^ groups (**Figure 1C**). These findings suggest that PBIS can serve as both a predictive and prognostic biomarker for AB treatment.

### PBIS as an independent biomarker in AB treatment for HCC

We further validated whether the PBIS could independently predict the clinical outcomes of AB treatment (**Table 3**). Multivariate logistic regression analysis showed that, among the various clinical factors, PBIS^hi^ and NLR were the factors associated with a poorer ORR. In multivariate Cox regression analyses, AFP, ECOG performance status, Child-Pugh score, and intrahepatic tumor size, PBIS^hi^, NLR, and CRP were associated with OS. Additionally, previous treatment history, viral etiology, AFP and PIVKA-II levels, ECOG performance status, multiple intrahepatic tumors, largest intrahepatic tumor size, PBIS^hi^, NLR, and CRP were related to PFS.

**Table 3:** Multivariate logistic regression and Cox-regressions analyzing associations between ORR, OS or PFS and each baseline characteristics.

	ORR		OS		PFS	
	OR (95% CI)	p	HR (95% CI)	p	HR (95% CI)	p
Gender	3.14 (0.94-14.47)	0.091	not included		not included	
Age	not included		not included		not included	
Treatment experienced	not included		not included		3.08 (1.72-5.52)	<.0001
Viral etiology	not included		not included		1.81 (1.03-3.17)	0.038
AFP	not included		1.00 (1.00-1.00)	0.001	1.00 (1.00-1.00)	0.003
PIVKA-II	1.00 (1.00-1.00)	0.334	1.00 (1.00-1.00)	0.055	1.00 (1.00-1.00)	0.003
ECOG	not included		2.56 (1.32-4.95)	0.005	1.82 (1.13-2.94)	0.014
Child-Pugh score	not included		1.44 (1.05-1.96)	0.022	not included	
Largest intrahepatic tumor	not included		0.92 (0.84-0.99)	0.036	not included	
Multiple intrahepatic tumor	not included		2.41 (0.96-6.03)	0.061	1.56 (0.89-2.73)	0.117
PVTT	not included		not included		not included	
EHS	not included		not included		1.60 (0.98-2.61)	0.06
**High PBIS**	**0.39 (0.15-0.94)**	**0.039**	**3.47 (1.51-7.96)**	**0.003**	**2.40 (1.41-4.09)**	**0.001**
**NLR**	0.62 (0.31-1.25)	0.183	**2.08 (1.12-3.85)**	**0.020**	**1.72 (1.04-2.83)**	**0.034**
**CRP**	**0.48 (0.23-0.98)**	**0.045**	**2.65 (1.33-5.27)**	**0.006**	**1.88 (1.15-3.07)**	**0.012**

ORR, objective response rate; OS, overall survival; PFS, progression-free survival; OR, odds ratio; CI, confidence interal; HR, hazard ratio; AFP, alpha-fetoprotein; PIVKA-II, protein induced by vitamin K antagonist-II; ECOG, Eastern Cooperative Oncology Group; PVTT, portal vein tumor thrombosis; EHS, extrahepatic spread; PBIS, peripheral blood inflammatory score; NLR, neutrophil-lymphocyte ratio; CRP, c-reactive protein. Bold values indicate the lowest p-values for OS and PFS, which we defined high peripheral blood inflammatory score.

In detail, PBIS demonstrated superior predictive performance for OS (HR 3.47, p=0.003), PFS (HR 2.40, p=0.001), and ORR (OR 0.39, p=0.039) compared to CRAFITY (OS: HR 2.15, p=0.026; PFS: HR 1.78, p=0.027), NLR alone (OS: HR 2.08, p=0.020; PFS: HR 1.72, p=0.034), and CRP alone (OS: HR 2.65, p=0.006; PFS: HR 1.88, p=0.012). Regarding the cytokine components, removing IL-2 slightly reduced the score’s performance (OS: HR 3.12, p=0.005; PFS: HR 2.21, p=0.004), while removing IL-12 had a more noticeable impact (OS: HR 2.84, p=0.008; PFS: HR 2.05, p=0.007). Using only NLR+CRP further decreased predictive value (OS: HR 2.54, p=0.007; PFS: HR 1.83, p=0.015) (**Table 3**, **Supplementary Table S3**). These findings suggest that while individual cytokine effects are modest, their inclusion significantly enhances the overall prognostic value of our composite PBIS score.

**Figure 2:**
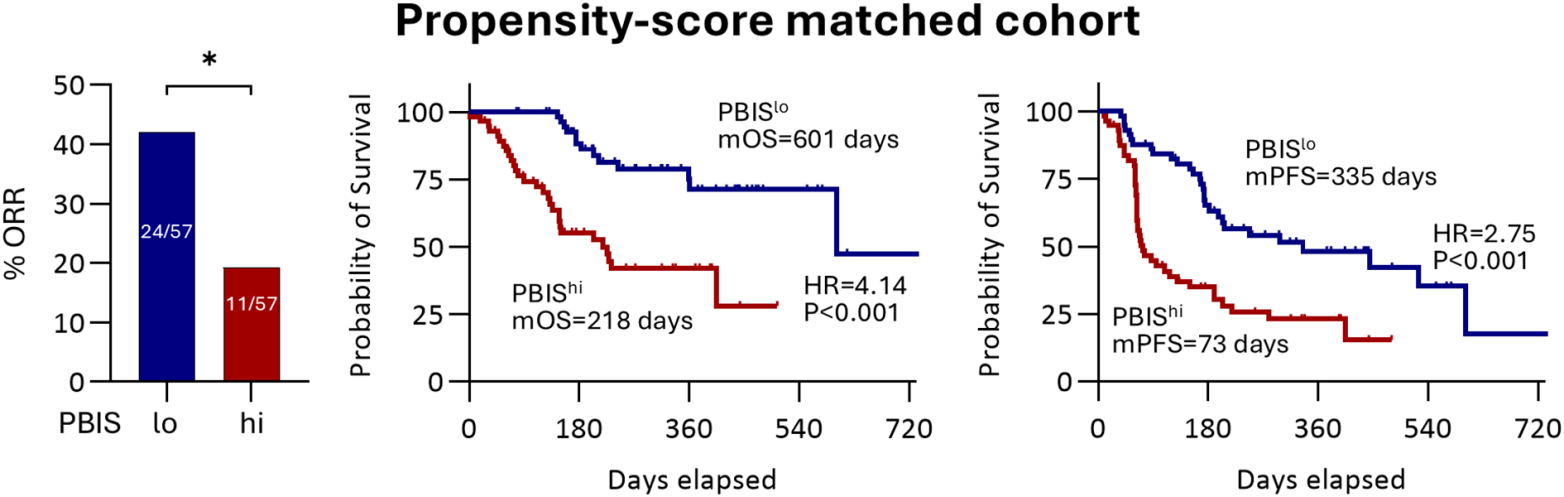
Clinical significance of PBIS in the propensity score-matched cohort. The percentage of ORR and survival curves regarding OS and PFS comparing PBIS^lo^ and PBIS^hi^ groups (n = 57, respectively). ORR, objective response rate; PBIS, peripheral blood inflammatory score; mOS, median overall survival; HR, hazard ratio; mPFS, median progression-free survival. * p < 0.05.

Finally, we performed 1:1 propensity score matching between the PBIS^hi^ and PBIS^lo^ groups (n = 57), and the factors between the two groups were well matched (**Supplementary Table S4**). In line with data from the unmatched cohort, the PBIS^hi^ group showed a significantly lower ORR than that in the PBIS^lo^ group (11/57, 19.3% vs. 24/57, 42.1%, p = 0.014) (**Figure 2**). In addition, the PBIS^hi^ group had a poorer OS (HR = 4.14, p<0.001) and PFS (HR = 2.75, p<0.001) than that in the PBIS^lo^ group (**Figure 2**). These findings suggest that PBIS is a reliable biomarker for the AB treatment of HCC.

## Discussion

In this prospective study of 170 patients treated with AB for HCC, we examined the relationship between clinical outcomes and baseline peripheral blood markers, including serum cytokines, measured by cytokine multiplex assay. Among various peripheral blood inflammatory cytokines, patients with high IL-2 and IL-12 levels showed poor OS and PFS after AB treatment. By including two known peripheral markers, NLR and CRP, we made PBIS that showed a strong association between PBIS and clinical outcomes. Through sensitivity analysis, we defined high PBIS as elevated levels of two or more of these markers. The high PBIS group had significantly poorer OS, PFS, and ORR in both the derivation and validation cohorts, which was distinct from the lenvatinib-treated cohort. In the propensity score-matched cohort, the PBIS^hi^ group showed significant associations with poor OS, PFS, and ORR, suggesting its robustness as a biomarker of AB treatment for HCC. The strength of our analysis is that we identified the relationship between IL-2 and IL-12 and the clinical outcomes of AB treatment in patients with unresectable HCC for the first time with a relatively large sample size compared to previous studies. We integrated these findings with well-known peripheral blood biomarkers such as NLR and CRP levels to develop a scoring model with stronger predictive power. Additionally, we validated these findings using an independent external cohort and propensity score matching, which provided confidence in this marker.

Immunotherapy has made significant strides and is applied at the forefront of cancer treatment, especially for HCC. (15, 16) However, the lack of reliable biomarkers for predicting clinical responses is a limiting factor for maximizing the potential of immunotherapy. Cellular stress, injury, infection, and inflammation result in the production and release of cytokines. These cytokines play crucial roles in regulating immune cell activity and influence all stages of carcinogenesis. This close association suggests that cytokines have the potential to serve as biomarkers for HCC treatment. A recent study of 64 patients treated with AB for HCC identified serum IL-6 as a biomarker. (17) The study showed that high levels of plasma IL-6 were significantly correlated with a low response rate and shorter PFS and OS. (17) This finding was further validated by another study that demonstrated that elevated serum IL-6 was associated with diminished clinical benefits of AB treatment, which might be related to the suppression of T-cell responses. (10) However, our study demonstrated that IL-6 levels were not associated with clinical outcomes, warranting further validation of these findings in future studies. We observed that the PBIS^hi^ group showed an overall increase in serum inflammatory cytokine levels, including IL-6, suggesting that systemic inflammation itself might contribute to the decreased effect of immunotherapy for HCC.

Markers of systemic inflammation have been recognized as predictive indicators of the efficacy of immunotherapy in HCC, with the NLR and CRP being notable biomarkers. (8) NLR is the ratio of neutrophil and lymphocyte counts in the peripheral blood, representing the innate and adaptive immune systems. (18) High NLR is associated with poor clinical outcomes in patients treated with AB combination therapy for HCC. (8) Another marker, CRP, has been studied in HCC because of its association with inflammation in carcinogenesis. (19) When combined with AFP level for a scoring system, as the “CRAFITY” score, AB-treated patients with HCC and lower scores showed better OS, PFS, and response. (20) Instead of AFP, we have added serum cytokines including IL-2 and IL-12 in addition to the NLR and CRP, and the combined scoring system significantly also predicted OS, PFS, and response, suggesting that measuring baseline cytokines at pretreatment should be considered in future biomarker studies.

In comparing our PBIS with other prognostic biomarkers, PBIS demonstrated a markedly enhanced or complementary ability to predict treatment outcomes (ORR, PFS, OS) in unresectable HCC patients receiving AB. Established indices like the CRAFITY score (combining CRP and AFP) and simpler single markers such as the NLR or CRP each show significant prognostic value, but PBIS’s multi-dimensional approach appears to outperform them. Notably, PBIS outperformed both CRAFITY and NLR alone in our cohort, demonstrating a stronger association with treatment outcomes. For example, high PBIS was associated with a markedly higher risk of death and disease progression than indicated by CRAFITY or NLR (HRs for OS and PFS nearly ~1.5–2-fold greater). PBIS-high patients (≥2 factors elevated) in our cohort achieved an ORR of only ~17–18% vs. ~37% in PBIS-low counterparts and had significantly worse survival (HR for OS ~3.6–4.0; HR for PFS ~2.1–4.6 for high vs. low), exceeding the prognostic separation afforded by NLR or CRAFITY alone. CRAFITY adds a tumor burden component (AFP) to inflammation (CRP), improving on single markers. PBIS, in turn, builds upon these concepts by simultaneously accounting for innate immune imbalance (NLR), systemic inflammatory response (CRP), and key immune cytokine signals (IL-2, IL-12). By integrating these diverse biomarkers, PBIS provides a more holistic reflection of a patient’s inflammatory and immune status. This multi-dimensional approach yields greater prognostic discrimination than any single parameter – PBIS-high status remained an independent predictor of inferior ORR, PFS, and OS in multivariate analyses even after accounting for clinicopathologic factors and outperformed other blood-based scores in direct comparisons. Therefore, while CRAFITY and NLR offer valuable predictive insight into AB treatment outcomes, the PBIS complements and surpasses these markers by capturing the convergent influence of systemic inflammation, thereby more reliably stratifying patients’ likelihood of response and survival

IL-2 is a key cytokine in cancer surveillance, is involved in innate and adaptive immunity, and plays a pivotal role in the proliferation of natural killer (NK) cells and T lymphocytes. (12, 21) The anti-tumoral effect of IL-2 is manifested by activation of NK cells and CD8^+^ T cells through binding to the low-affinity IL-2 receptor. (22) However, IL-2 exhibits an immunosuppressive role as it induces the activation of regulatory T (T_reg_) cells, which express a high-affinity IL-2 receptor, resulting in a pro-tumoral effect. (22, 23) Stimulated adjacent T_reg_ cells induce cytokine-deprivation-mediated apoptosis in effector T cells, which cells play a crucial role in cancer immunotherapy, resulting in a decreased systemic immune response that could contribute to disease progression. (24) A previous study demonstrated that a high T_reg_ cell-to-effector T cell ratio was associated with a decreased clinical response in AB-treated patients with advanced HCC. (25) A previous study demonstrated the possible prognostic value of elevated serum IL-2 levels in gastrointestinal cancers, presenting with poor mortality, which is consistent with our findings. (26) The relationship between elevated IL-2 levels and its immunosuppressive role in HCC immunotherapy requires further investigation.

IL-12 is a pro-inflammatory cytokine that stimulates NK cell and CD8^+^ T cell proliferation, along with promoting helper T cell differentiation and its antigen presentation. (23) In addition, IL-12 has anti-angiogenic properties, making it an optimal target for cancer therapy. (23) However, IL-12 is thought to play a major role in the systemic inflammatory response. (27) Although it has been generally considered to have antitumor effects, baseline elevation of this cytokine may reflect systemic inflammation, which can limit the augmentation of antitumor T-cell responses by immunotherapy in patients with HCC.

In conclusion, we showed that the PBIS scoring system using serum IL-2 and IL-12 levels in addition to the NLR and CRP analyzed at the baseline of AB treatment in patients with HCC can be a competent prognostic and predictive biomarker for clinical outcomes. This non-invasive blood-based marker could be validated in a larger international cohort.

## Data Availability

The original contributions presented in the study are included in the article/**Supplementary Material**. Further inquiries can be directed to the corresponding author.
