# Supplementary material for: Peripheral blood inflammatory score using a cytokine multiplex assay predicts clinical outcomes in patients treated with atezolizumab-bevacizumab for unresectable HCC
Source: Front Immunol. 2025 Jun 11;16:1578422. doi: 10.3389/fimmu.2025.1578422 (PMC12187687; doi:10.3389/fimmu.2025.1578422)
Supplement: Supplementary file 1 [file DataSheet1.pdf]

Supplementary Table S1. Univariate Cox-regressions analyzing associations between OS or PFS and peripheral inflammatory markers.

	OS		PFS	
	HR (95% CI)	p	HR (95% CI)	p
**NLR**	**1.27 (1.16-1.39)**	**< .0001**	1.16 (1.06-1.27)	**0.001**
**CRP**	**1.14 (1.04-1.24)**	**0.004**	1.09 (1.01-1.16)	**0.018**
IFN-γ	1.00 (1.00-1.00)	0.408	1.00 (1.00-1.00)	0.14
IL-10	1.00 (1.00-1.01)	0.279	1.00 (1.00-1.01)	0.261
**IL-12**	**1.01 (1.00-1.01)**	**0.002**	1.00 (1.00-1.01)	**0.013**
IL-17	1.00 (1.00-1.01)	0.159	1.00 (1.00-1.01)	0.058
**IL-2**	**1.01 (1.00-1.02)**	**0.046**	1.01 (1.00-1.02)	**0.009**
IL-6	1.01 (0.99-1.02)	0.279	1.00 (0.99-1.01)	0.473
TNF	1.01 (1.00-1.02)	0.052	1.01 (1.00-1.01)	0.12

OS, overall survival; PFS, progression-free survival; HR, Hazard Ratio; CI, Confidence Interval; NLR, Neutrophil-to-Lymphocyte Ratio; CRP, C-Reactive Protein; IFN-γ, Interferon Gamma; IL-10, Interleukin 10; IL-12, Interleukin 12; IL-17, Interleukin 17; IL-2, Interleukin 2; IL-6, Interleukin 6; TNF, Tumor Necrosis Factor.

Supplementary Table S2. Comparison of characteristics between PBIS high and low groups in each cohort.

	Derivation Cohort			Validation Cohort			Lenvatinib Cohort		
	PBIS^{lo} (n=54)	PBIS^{hi} (n=62)	p	PBIS^{lo} (n=15)	PBIS^{hi} (n=39)	p	PBIS^{lo} (n=11)	PBIS^{hi} (n=10)	p
Male gender	42 (77.8%)	51 (82.3%)	0.531	13 (86.7%)	33 (84.6%)	0.841	8 (72.7%)	8 (80.0%)	0.711
Age, years	63.6 ± 12.1	61.8 ± 12.5	0.451	64.3 ± 8.9	64.1 ± 11.9	0.955	63.4 ± 9.5	63.1 ± 10.8	0.946
Treatment history	37 (68.5%)	33 (53.2%)	0.089	8 (53.3%)	22 (56.4%)	0.836	6 (54.5%)	7 (70.0%)	0.461
Viral etiology	39 (72.2%)	42 (67.7%)	0.598	14 (93.3%)	30 (76.9%)	0.161	9 (81.8%)	7 (70.0%)	0.532
AFP, ng/mL	8244.1 ± 17436.9	20298.5 ± 39918.8	0.038	3039.4 ± 6011.7	19186.9 ± 48892.5	0.045	3954.2 ± 5421.3	15687.3 ± 22436.2	0.034
PIVKA-II, mAU/mL	18453.8 ± 78137.8	22670.2 ± 46096.1	0.723	11534.0 ± 13076.5	7325.6 ± 10029.3	0.268	9087.5 ± 11546.1	13454.8 ± 14652.7	0.436
ECOG 0	40 (74.1%)	36 (58.1%)	0.068	12 (80.0%)	26 (66.7%)	0.333	10 (90.9%)	8 (80.0%)	0.459
Child B	4 (7.4%)	11 (17.7%)	0.095	0 (0.0%)	0 (0.0%)	-	0 (0.0%)	0 (0.0%)	-
Largest tumor size, cm	5.0 ± 4.8	8.9 ± 5.8	<0.001	8.3 ± 4.8	8.6 ± 6.0	0.864	7.1 ± 5.2	8.3 ± 4.9	0.589
Multiple tumors	32 (59.3%)	50 (80.6%)	0.011	14 (93.3%)	36 (92.3%)	0.893	9 (81.8%)	9 (90.0%)	0.594
PVTT	29 (53.7%)	41 (66.1%)	0.167	9 (60.0%)	29 (74.4%)	0.298	6 (54.5%)	6 (60.0%)	0.803
EHS	28 (51.9%)	33 (53.2%)	0.882	6 (40.0%)	23 (59.0%)	0.208	5 (45.5%)	6 (60.0%)	0.51

Values are presented as mean ± standard deviation or number (percentage). AFP: alpha-fetoprotein; PIVKA-II: protein induced by vitamin K absence or antagonist-II; ECOG: Eastern Cooperative Oncology Group; PVTT: portal vein tumor thrombosis; EHS: extrahepatic spread.

Supplementary Table S3. Multivariate logistic regression and Cox-regressions analyzing associations between ORR, OS or PFS and each baseline characteristics.

	ORR		OS		PFS	
	OR (95% CI)	p	HR (95% CI)	p	HR (95% CI)	p
CRAFITY	0.56 (0.27-1.15)	0.115	**2.15 (1.10-4.23)**	**0.026**	**1.78 (1.07-2.94)**	**0.027**
**PBIS (complete)**	**0.39 (0.15-0.94)**	**0.039**	**3.47 (1.51-7.96)**	**0.003**	**2.40 (1.41-4.09)**	**0.001**
PBIS without IL-2	0.43 (0.18-1.02)	0.056	**3.12 (1.42-6.87)**	**0.005**	**2.21 (1.30-3.78)**	**0.004**
PBIS without IL-12	0.45 (0.19-1.07)	0.072	**2.84 (1.32-6.15)**	**0.008**	**2.05 (1.21-3.46)**	**0.007**
PBIS with NLR+CRP only	0.52 (0.25-1.10)	0.086	**2.54 (1.28-5.02)**	**0.007**	**1.83 (1.12-2.98)**	**0.015**

ORR, Objective Response Rate; OR, Odds Ratio; OS, Overall Survival; HR, Hazard Ratio; PFS, Progression-Free Survival; CI, Confidence Interval; PBIS, Peripheral Blood Inflammatory Score; IL-2, Interleukin-2; IL-12, Interleukin-12; CRP and AFP in immunotherapy.

Supplementary Table S4. Baseline characteristics before and after propensity-score matching.

	Before matching			After matching		
	$PBIS^{lo}$ (n=69)	$PBIS^{hi}$ (n=101)	p	$PBIS^{lo}$ (n=57)	$PBIS^{hi}$ (n=57)	p
Male gender	55 (79.7%)	84 (83.2%)	0.711	44 (77.2%)	44 (77.2%)	1
Age, years	63.7 ± 11.6	62.7 ± 12.4	0.585	64.1 ± 12.3	64.4 ± 11.5	0.896
Treatment history	45 (65.2%)	55 (54.5%)	0.214	35 (61.4%)	35 (61.4%)	1
Viral etiology	53 (76.8%)	72 (71.3%)	0.532	42 (73.7%)	42 (73.7%)	1
AFP, ng/mL	7112.6 ± 15940.4	19869.3 ± 43824.2	0.008	8533.0 ± 17220.3	8675.1 ± 16512.2	0.964
PIVKA-II, mAU/mL	16949.5 ± 69960.6	16745.0 ± 37590.0	0.982	11069.4 ± 34751.7	16449.7 ± 38650.7	0.436
ECOG 0	52 (75.4%)	62 (61.4%)	0.089	40 (70.2%)	37 (64.9%)	0.541
Child B	4 (5.8%)	11 (10.9%)	0.382	4 (7.0%)	5 (8.8%)	1
Largest tumor size, cm	5.7 ± 5.0	8.8 ± 5.9	<0.001	6.4 ± 4.7	7.4 ± 5.7	0.277
Multiple tumors	46 (66.7%)	86 (85.1%)	0.008	44 (77.2%)	45 (78.9%)	1
PVTT	38 (55.1%)	70 (69.3%)	0.083	36 (63.2%)	36 (63.2%)	1
EHS	34 (49.3%)	56 (55.4%)	0.525	25 (43.9%)	28 (49.1%)	0.707

Values are presented as mean ± standard deviation or number (percentage). AFP: alpha-fetoprotein; PIVKA-II: protein induced by vitamin K absence or antagonist-II; ECOG: Eastern Cooperative Oncology Group; PVTT: portal vein tumor thrombosis; EHS: extrahepatic spread.

Supplementary Figure S1. Correlation coefficients between serum cytokine concentrations and baseline characteristics.

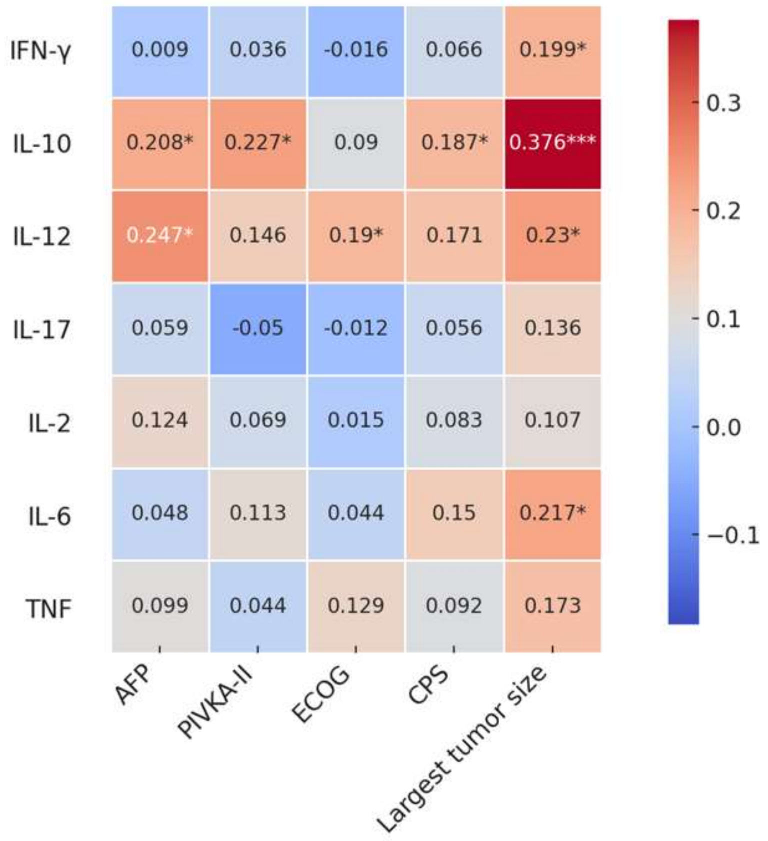

Data are presented as Spearman correlation coefficients. *p<0.05; **p<0.01; ***p<0.001. AFP, alpha-fetoprotein; PIVKA-II, protein induced by vitamin K antagonist-II; ECOG, Eastern Cooperative Oncology Group; CPS, Child-Pugh score; PVTT, portal vein tumor thrombosis; EHS, extrahepatic spread; IFN-γ: Interferon-gamma; IL-10: Interleukin-10; IL-12: Interleukin-12; IL-17: Interleukin-17; IL-2: Interleukin-2; IL-6: Interleukin-6; TNF: tumor necrosis factor.

Supplementary Figure S2. Scatter plots showing correlations between inflammatory cytokines and clinical features in the derivation cohort.

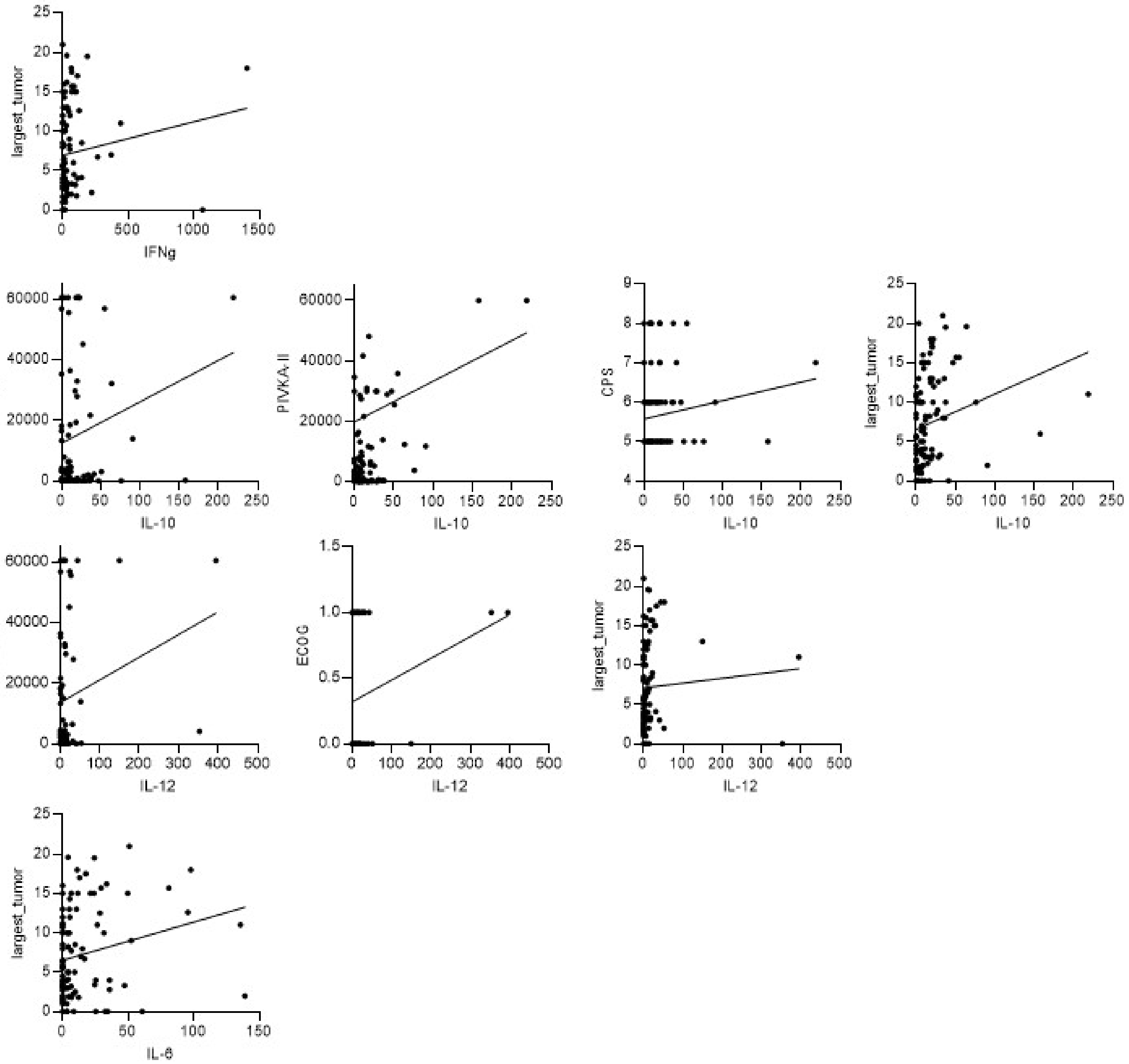

AFP, alpha-fetoprotein; PIVKA-II, protein induced by vitamin K antagonist-II; ECOG, Eastern Cooperative Oncology Group; CPS, Child-Pugh score; IFN-g, Interferon-gamma; IL-10, Interleukin-10; IL-12, Interleukin-12; IL-6, Interleukin-6.

Supplementary Figure S3. Comparison of serum cytokine levels between PBIS low (n=69) and high (n=101) groups.

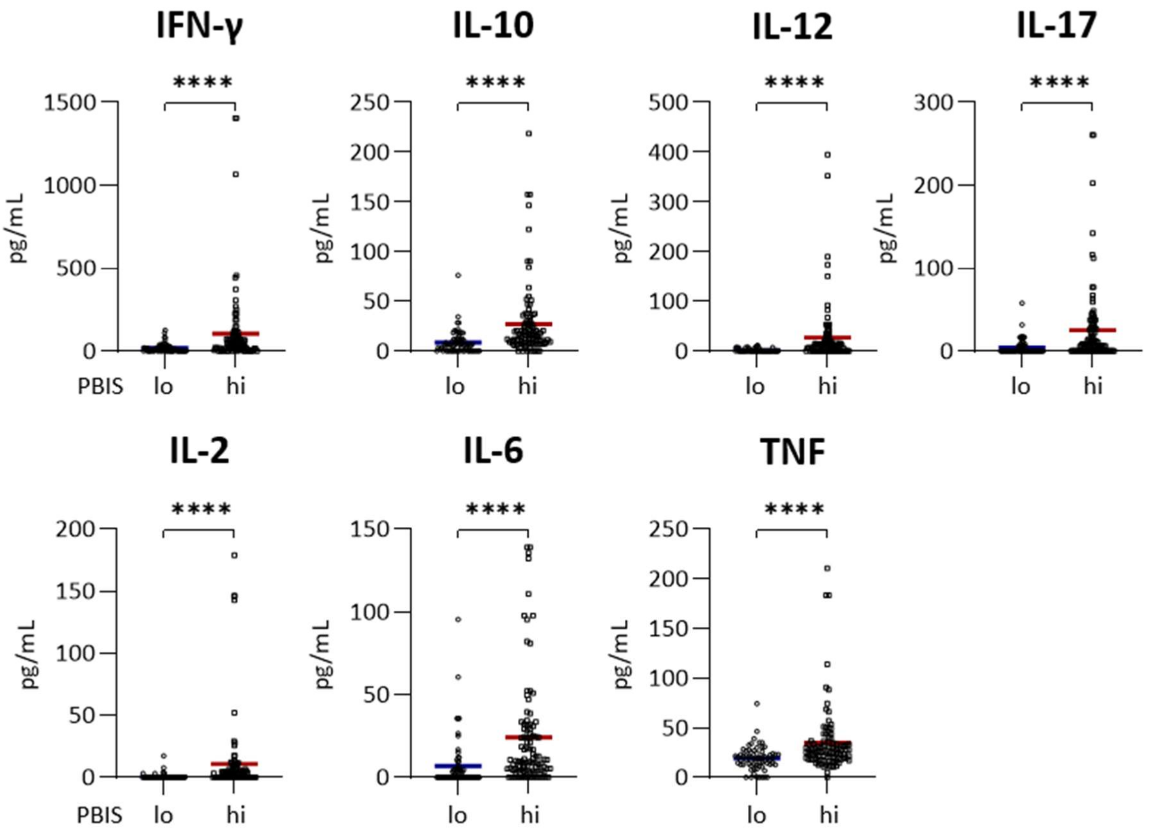

PBIS, peripheral blood inflammatory score; IFN-γ: Interferon-gamma; IL-10: Interleukin-10; IL-12: Interleukin-12; IL-17: Interleukin-17; IL-2: Interleukin-2; IL-6: Interleukin-6; TNF: tumor necrosis factor. ****$p<0.0001$.
